# A Fair Performance Comparison between Complex-Valued and Real-Valued Neural Networks for Disease Detection

**DOI:** 10.3390/diagnostics12081893

**Published:** 2022-08-04

**Authors:** Mario Jojoa, Begonya Garcia-Zapirain, Winston Percybrooks

**Affiliations:** 1 Department of Electrical and Electronics Engineering, University of North, Barranquilla 080002, Colombia; 2 Department of Computer Science and Engineering, University of Deusto, 48007 Bilbao, Spain

**Keywords:** complex-valued deep learning, complex-valued convolution neural networks, real-valued neural networks, complex numbers, fair performance comparison

## Abstract

Our aim is to contribute to the classification of anomalous patterns in biosignals using this novel approach. We specifically focus on melanoma and heart murmurs. We use a comparative study of two convolution networks in the Complex and Real numerical domains. The idea is to obtain a powerful approach for building portable systems for early disease detection. Two similar algorithmic structures were chosen so that there is no bias determined by the number of parameters to train. Three clinical data sets, ISIC2017, PH2, and Pascal, were used to carry out the experiments. Mean comparison hypothesis tests were performed to ensure statistical objectivity in the conclusions. In all cases, complex-valued networks presented a superior performance for the Precision, Recall, F1 Score, Accuracy, and Specificity metrics in the detection of associated anomalies. The best complex number-based classifier obtained in the Receiving Operating Characteristic (ROC) space presents a Euclidean distance of 0.26127 with respect to the ideal classifier, as opposed to the best real number-based classifier, whose Euclidean distance to the ideal is 0.36022 for the same task of melanoma detection. The 27.46% superiority in this metric, as in the others reported in this work, suggests that complex-valued networks have a greater ability to extract features for more efficient discrimination in the dataset.

## 1. Introduction

Deep learning has marked a milestone in every field of science. Its application in medicine has spread quickly in recent years. Progress has been made from the augmented intelligence view [1], in which efforts focus on increasing the doctor’s capacity to detect pathology patterns that are not easily visible to the human eye. Hence, developing algorithms that perform well is very important for the scientific community. Developing new structures is necessary to obtain better results to collaborate in disease detection with high confidence. Deep learning structures based on complex numbers [2] have gradually been developed; however, the theoretical mathematic support is limited, slowing the rapid evolution of algorithms of this type. On the other hand, in recent years, we have found works [3,4,5,6] with these new deep learning models being addressed in applications in which the input data maintain the information in magnitude and phase. This suggests that these novel algorithms have a greater ability to use the information analyzed [7,8]. Furthermore, we can generically express that complex-valued deep learning (CVDL) is a higher level of real-valued deep learning, as the latter can be considered a particular case when the imaginary part of the CVDL is zero. We have, therefore, focused on a fair comparison of real-value-based and complex-value-based classification structures to discover which one shows the best performance for the same task under similar conditions. It is important to highlight that the use of data in the real number domain at the input does not affect the performance of the structures being studied. Instead, this condition enables the observation of how the algorithms behave for raw inputs in the real numerical domain. Therefore, this data must be appropriately represented to perform operations in complex numbers, i.e., with a real component and an imaginary one. For this purpose, we chose a Fourier transformation stage via the symmetric Vandermonde matrix [9], which uses Hermitian symmetry to reduce the number of components by almost half. Additionally, the Fourier transform maintains the information of magnitude and frequency of the input signal, represented in the complex-number domain. On the other hand, the algorithm Fast Fourier Transform is very efficient with low computational cost [10].

This approach has the aim to reduce the possible biases that can hide the interpretation [11] of the results related to the greater capacity of the complex-valued structure compared with the real-valued ones. We chose three clinical datasets to achieve this goal. This enabled us to observe the behavior of the algorithms studied from an objective point of view. The following metrics were proposed for this study: F1 Score, Precision, Recall/Sensitivity, Accuracy, and Specificity. For all the cases, the structure/algorithm based on complex numbers showed better performance on average. We managed to check this through a hypothesis test (Student’s *t*-test for F1 Score, Precision, Recall, and Specificity and Mann–Whitney U test for Accuracy) to compare the means of each metric obtained for both studied structures. The main contribution of this research work is the demonstration of the higher capacity of the complex-valued deep learning structures to solve health care data classification problems compared with their homologous real-valued structures ones. In addition, our work opens the door to building more efficient convolutional networks in the complex-number domain for health (melanoma and heart murmurs) real-valued input data since CVDL performs better than RVDL using a similar number of trainable parameters.

Medical work requires high reliability and precision. Therefore, if we want to build a digital tool that helps health professionals detect diseases automatically, it is necessary to obtain deep learning classification models with high performance for the proposed tasks. It is true that current artificial intelligence algorithms are solutions to these problems with outstanding metrics. In 2021 we presented the use of Mask RCNN and ResNet 152 [12] for melanoma detection in dermoscopic signals. However, we showed that the performance of the real-valued algorithms is proportional to their computational complexity in terms of depth of structure, number of trainable parameters, and amount of training data. This leads to structural limitations and limitations in achieving convolutional networks with superior performance. For this reason, we decided to study complex-valued deep learning structures to test their performance compared to real-valued deep learning structures and thereby demonstrate that, for real-valued inputs, complex-valued algorithms have superior discriminative performance for the disease detection task under equivalent operating conditions. We concluded that with an equal number of trainable parameters, more powerful and high-performing classification complex-valued structures can be obtained. The next step is bringing these structures to production applications. The latter can be achieved by applying transfer learning techniques to train the complex-valued algorithms.

This work is divided into seven sections, as described below. Section 1 and Section 2, in which we give a brief description of the datasets used and an exhaustive review of the state of the art, Section 3, in which we give an in-depth description of the structures designed, experiment design, and hypothesis test, Section 4, with details on the results obtained with the metrics proposed, and we address the strengths and weaknesses detected in this research work. Additionally, in this section, we describe the boundaries of the results obtained, and lastly, Section 5, in which we describe the knowledge generated through the development of this research.

### Review of the State of the Art of the Technique

To contribute to this branch of science, we carried out an exhaustive review of works related to the use of complex-valued convolution neural networks. The following articles are noteworthy for their contribution to the state of the art. It is remarkable that no applications of this type of network were found in datasets consisting of images of melanoma or scalograms of heart sounds. Table 1 below shows a summary of the review of works related to the area of study.

As can be observed, complex-valued neural networks have been applied in health as well as other fields. In [8], the authors propose the use of a complex-valued neural network to classify complex fMRI data. They reached high accuracies, but they did not perform a comparison of their results with similar real-valued neural networks, and they did not use real-valued input data for their experiments. In [13], the authors used the CVDL to predict the glucose concentration estimation with a Mean Square Error (MSE) of 0.011. They did not study the behavior of this network for classification problems. In [3], the authors carried out a state-of-the-art study to show that complex-valued neural networks have special characteristics that make them powerful, while they underscore that their properties must be explored in much greater depth to use the ability of these algorithms that are emerging in deep learning. In [4,5,6,14], the authors proposed different applications of the CVDL, such as motion estimation, in-SAR and SAR radar complex data classification, channel state information for high-performance decode tasks, and others. However, they did not compare the performance between complex-valued and equivalent real-valued algorithms. In [4], the use of Fast Fourier Transform (FFT) is proposed to represent the data in the complex numbers domain. On the other hand, in [7], X-ray chest images were denoised using complex-valued neural networks, showing the high capacity of this kind of structure in health applications. In [15,16,17,18], the authors used the CVDL and complex-valued data to detect brain diseases using fMRI data, reaching outstanding performance. The key differentiating factor of this work compared with our proposed one is the nature of the input data, because we are using skin images and scalograms built from heart sounds. Moreover, they did not use raw real-valued data as input, and they did not compare the results with similar real-valued homologs structures. Based on the above, we have focused on our efforts to demonstrate the better performance of complex-valued deep learning compared with real-valued deep learning to solve real-valued health data classification problems. To carry out a fair comparison, we have used a similar number of trainable parameters to clarify that the power of these new algorithms is the consequence of the complex-number nature and not of the difference in the number of trainable parameters.

## 2. Materials

To achieve the objective of comparing complex-valued and real-valued structures, three clinical datasets of different types have been selected; ISIC2017 and PH2, related to melanoma pathology, and Pascal, associated with heart sounds. They are described below.

### 2.1. ISIC2017

**Figure 1:**
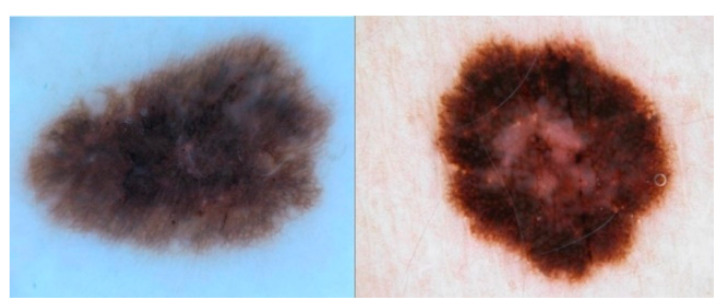
Examples of normal and abnormal images from the ISIC2017 dataset.

The dataset is composed of 1995 images for deep learning analysis [19,20]. This dataset was published with a challenge [21] to researchers across the world to join forces to achieve good enough performance metrics to bring these models into production. This dataset is available at https://challenge.isic-archive.com/data/ (accessed on 1 February 2022). Figure 1 shows an example of the images contained in the dataset.

### 2.2. PH2

The increase in cases of melanoma [22] has recently prompted the development of computer-assisted diagnostic systems to classify dermatoscopy images [23]. Fortunately, the performance of such systems can be compared, as they can be evaluated on different sets of images. Public databases are available to make a fair assessment of multiple systems. We chose to use the PH2 dermatoscopy image database for this research. It includes manual segmentation, clinical diagnosis, and identification of several dermatoscopy structures performed by expert dermatologists on a set of 200 images. The PH2 database is available free of charge for research and benchmarking purposes [24]. It can be accessed at https://www.fc.up.pt/addi/ph2%20database.html, (accessed on 1 February 2022) Figure 2 shows an example of the images contained in the dataset.

### 2.3. PASCAL

**Figure 3:**
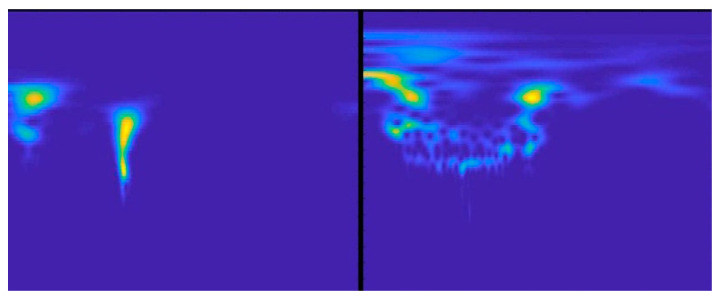
Images of a normal and abnormal scalogram retrieved from the PASCAL database.

The PASCAL database comprises 461 recordings for the classification of heart sounds. Of these, 320 are normal sounds and 141 are abnormal/pathological sounds. Although the number of recordings is relatively large, the author’s version of the article published in *Physiological Measurement* [25] describes that the recordings last from 1 to 30 s. They also have a limited frequency range, under 195 Hz, due to the low pass filter applied. See Ph2 at www.peterjbentley.com/heartchallenge (accessed on 1 February 2022). Figure 3 shows an example of the scalograms obtained with the sounds from the dataset.

Table 2, shown below, is a summary of the relevant characteristics of the datasets used in this research work.

## 3. Methods

**Figure 4:**
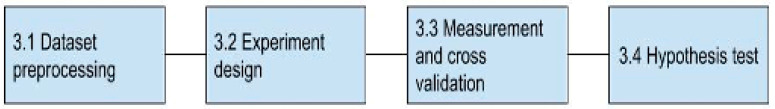
Block diagram of the proposed solution approach.

Figure 4 shows the block diagram with the steps performed to achieve the research objective.

### 3.1. Dataset Preprocessing

The images were scaled to a standard dimension of 224 × 224 pixels and normalized based on the mean and standard deviation of the sets of pixels that form them. Equation (1) describes an executed mathematical procedure.

(1) $$Img_{1}=\frac{Img_{0}-\mu \;}{desvStd\;\left(Img_{0}\right)}$$

Equation (1). Image centered with respect to the mean and normalization based on division by the standard deviation.

It is remarkable that this stage of normalization is executed in the training stage and in the testing stage with the same values to reduce the distortion caused by data normalization. Scalograms were chosen as inputs for heart sounds. We have applied algorithms for automatic trimming and to obtain the scalogram through the wavelet transform. The used methods were published by Jojoa et al. in [26].

### 3.2. Experiment Design

We found it necessary to design two-factor experiments in this stage of the research. The structure factor has two levels, which are complex-valued and real-valued. Moreover, the database factor has the ISIC2017, PH2, and PASCAL levels. Table 3 shows a summary of the experiment design.

The F1 Score, Precision, Recall, Accuracy, and Specificity metrics were calculated for each factor combination in all the datasets studied.

#### 3.2.1. Structure Factor

To make a fair comparison of the performance of real-valued and complex-valued networks, the design must be as similar as possible concerning the number of parameters and operations. This is achieved by obtaining two structures with the same layers and an equivalent number of trainable parameters.

#### 3.2.2. Complex-Valued Structure

We aim to run this algorithm to extract the largest amount of information from the inputs in all proposed experiments, in other words, from all the floating-point tensor inputs that may or may not be images [27]. If we limit these values to integers in the interval [0, 255], they will match an image. We, therefore, decided to use different types of signals to observe the behavior for the different cases. We assumed that a transformation in the numerical domain could find useful information to improve the classification capacity of a deep learning model. This would be achieved with dimensional improvement (increasing dimensions and/or components) of the class separability index since it would allow the drawing of decision regions that would better separate the classes involved and, thus, the performance of the system. Based on this, the convolution network design is carried out from a finite impulse response (FIR) filter approach, whose coefficients are learned and belong to the proposed numerical set. Equation (2) specifies the convolution process in this numerical domain.

(2) $$Z\left(x,y\right)*ZZ\left(z,y\right)=\overset{M-1}{\underset{x=0}{\sum }}\overset{N-1}{\underset{y=0}{\sum }}\left[R_{z}\left(m,n\right)+I_{z}\left(m,n\right)\right].\left[R_{zz}\left(x-m,y-n\right)+I_{zz}\left(x-m,y-n\right)\right]$$

Equation (2). Complex convolution between *Z* and *ZZ* complex functions.

It is noteworthy that the filters will execute operations in the complex number domain, whereby we have selected the Hilbert space as the space where the necessary complex operations will be performed [28]. It must also be considered that the dot product is the basis of forward propagation operations in deep learning. Furthermore, the average Pooling must [29] be defined in the numerical domain whenever it is the more intuitive that can be applied in the numerical set selected. The activation function that is used for this machine learning classification structure proposal is Complex Relu [26], which is described in Equation (3).

(3) $$g\left(z\right)=\left\{\begin{array}{c} z\;if\;R\left\{z\right\}>0\;and\;I\left\{z\right\}>0\\ 0,\;otherwise \end{array}\right\}$$

Equation (3). Complex Relu [17].

Once the basic operations needed to build the machine learning complex number-based structure have been defined, the input data must be converted from its original real numeric nature to the complex number’s domain. For this purpose, we decided to use the Fourier transform. However, to reduce the computational cost, we decided to use the Hermitian symmetry, which contains fewer parameters from the original Fourier matrix. This is shown in Equation (4).

(4) $$F=\begin{array}{c} \begin{array}{c} \begin{array}{c} w_{N}^{0,0}\\ w_{N}^{1,0}\\ \vdots \\ w_{N}^{\left(N-1\right),0} \end{array} \end{array} \end{array}\;\;\;\;\begin{array}{c} \begin{array}{c} \begin{array}{c} w_{N}^{0,1}\\ w_{N}^{1,1}\\ \vdots \\ w_{N}^{\left(N-1,0\right)} \end{array} \end{array} \end{array}\;\;\;\begin{array}{c} \begin{array}{c} \begin{array}{c} \cdots \\ \cdots \\ \ddots \\ \cdots \end{array} \end{array}\; \end{array}\;\begin{array}{c} \begin{array}{c} \begin{array}{c} w_{N}^{0,\left(N-1\right)}\\ w_{N}^{1,\left(N-1\right)}\\ \vdots \\ w_{N}^{\left(N-1,N-1\right)} \end{array} \end{array} \end{array},\;\;\;\;w_{N}=e^{-\frac{2k}{N}}$$

Equation (4). Vandermonde matrix from the Fourier transform.

**Figure 5:**
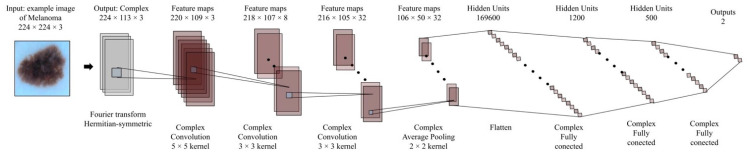
Convolution network structure based on complex numbers for this study.

All the above enable the possibility of a fair comparison of the results obtained. The convolution network structure in the complex number’s domain is shown in Figure 5.

**Figure 6:**
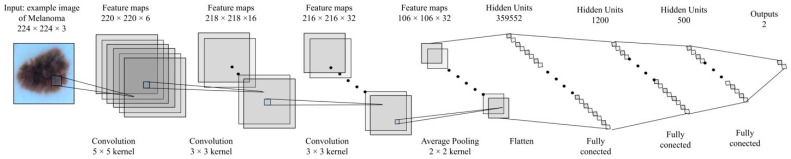
Convolution network structure based on real numbers for this study.

As can be observed, the network uses only convolutional layers, average Pooling, and Complex Relu. Based on this approach, we have built an equivalent convolution network in the real number’s domain. This is shown in Figure 6.

Furthermore, each one contains an equivalent number of parameters. In other words, correct conclusions are sought for the performance associated with the depth or the trainable number of parameters in the algorithms. Lastly, Table 4 shows the hyperparameters used for both structures involved in this study.

Similarly, Table 5 shows the number of trainable parameters per layer. Focusing on maintaining an equivalent structure approach, we have attempted to ensure that the complex-valued network maintains at least half as many parameters as the real-valued network. This was done to avoid the bias caused by the nature of two components, a real part and an imaginary part from the complex numbers.

### 3.3. Measurement and Cross-Validation

To compare the results obtained for each structure, we considered it necessary to measure several times in a repetitive manner in such a way that it allowed a comparative statistical analysis that eliminated subjectivity in the comparison process of the phenomenon. For this study, we decided to use a k folds cross-validation with k = 10 [30]. Based on theory, data normality and correlation tests should be performed [31] in order to apply a Student’s *t*-test for comparison of means.

### 3.4. Hypothesis Test

The comparison of the performance of the used metrics from a statistical approach is important in the scientific method. Hence, the two-tailed Student’s *t*-test [31] was chosen to observe whether there was sufficient statistical evidence to indicate that the means of the metrics calculated for the complex-valued networks are different from the means of the metrics calculated for the real-valued networks. Moreover, this test is used for its reliability with small samples [31].

Shapiro–Wilks [32]: The Student’s *t*-test is highly sensitive to data normality. As a consequence, it is necessary to apply data normality tests before executing the Student’s test. The Shapiro–Wilks test is based on the following hypotheses to be accepted or rejected, according to the *p*-value.

**H0:**  The data come from a normal distribution.

**H1:**  The data do not come from a normal distribution.

Student’s *t*-test: comparison [31]: It is important to perform a means comparison test to observe the differences from a statistical approach, reducing the subjectivity that may appear in the comparison procedure. We decided to use the Student’s *t*-test for F1, Precision, Recall, and Specificity mean metrics comparison. The Mann–Whitney U test was applied for Accuracy metric means comparison since this one did not accomplish the Shapiro–Wilks normality test. The hypotheses are accepted or rejected based on the *p*-value [32].

**H0:**  There is no statistical evidence to differentiate the means of the samples.

**H1:**  There is statistical evidence to differentiate the means of the samples.

We have used a confidence interval of 5% [31] for all the hypothesis tests in this study.

## 4. Results and Discussion

After building the convolution networks, we ran the experiments using Python 3.7 programming language, Complex-Pytorch framework development, and a GPU Nvidia RTX2080Ti. The code we used can be found in the following repository: https://github.com/mario42004/ComplexValuedDeepLearning, (accessed on 1 June 2022) We present the results for each of the case studies below. Table 6 shows the results obtained for 10 folds using the complex-valued convolution structure for melanoma detection in the set of dermatoscopy images from the ISIC2017 repository. We observe that the data meet the normality criteria to perform the Student’s *t*-test in all cases.

Table 7 shows the results obtained for 10 folds using the real-valued convolution structure for the task of melanoma detection in the set of dermatoscopy images from the ISIC2017 repository. We observe that the data satisfy the normality criteria to perform the Student’s *t*-test in all cases.

Table 8 shows the results obtained after having applied the Student’s *t*-test. We underscore that hypothesis H0 was rejected with a least 5% significance level.

Table 9 shows the results obtained for 10 folds using the complex-valued convolution structure for the task of melanoma detection. The data set of dermatoscopy images was the PH2 repository. We observe that the data satisfy the normality criteria to perform the Student’s *t*-test in almost all cases, except in Accuracy.

Table 10 shows the results obtained for 10 folds using the real-valued convolution structure for melanoma detection in the set of dermatoscopy images from the PH2 repository. We observe that the data satisfy the normality criteria to perform the Student’s *t*-test in almost all cases, except in Accuracy.

Table 11 shows the results obtained after having applied the Student’s *t*-test. It should be noted that hypothesis H0 was rejected with a least 5% significance level for all the samples of metrics obtained except Accuracy. We can thus conclude that the complex-valued convolution network performs better than its real counterpart for the metrics used in the classification task of dataset PH2.

Table 12 shows the results obtained for 10 folds using the complex-valued convolution structure for the task of detection of abnormality in the set of scalograms from the Pascal repository. We observe that the data satisfy the normality criteria to perform the later hypothesis test in almost all cases except for Accuracy.

Table 13 shows the results obtained for 10 folds using the real-valued convolution structure for the task of abnormality detection in the set of scalograms obtained with the Pascal repository. We observe that the data satisfy the normality criteria to perform the Student’s *t*-test in almost all cases, except in Accuracy. Table 11.

Table 14 shows the results obtained after having applied the Student’s *t*-test. It should be noted that hypothesis H0 was rejected with a least 5% significance level for all the samples of metrics obtained except Accuracy. We can thus conclude that the complex-valued convolution network performs better than its real counterpart for the metrics used in the classification task for the Pascal dataset.

**Figure 7:**
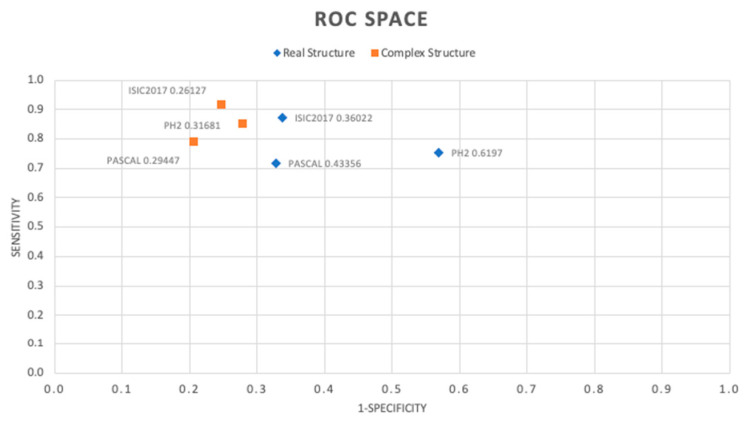
ROC space of the obtained classifier.

As can be seen, the Student’s *t*-test proves that the complex-valued network performs better, on average, for almost all the cases. For the Accuracy metric, the only one that did not accomplish the normality condition, we carried out the Mann–Whitney U test. The results are shown in Table 15 below:

Conversely, we decided to draw the specificity and sensitivity/recall metrics to observe the behavior of the classifiers obtained in the Figure 7 ROC space.

As can be seen, the classifiers in the complex number’s domain show a better performance. We achieved the best behavior in the ROC space for the classifier trained with the ISIC2017 dataset, with a Euclidean distance to the ideal coordinate classifier (0, 1) of 0.26127. Similarly, for the PH2 and Pascal datasets, better results were obtained with classifiers in the complex numbers domain, with distances of 0.31681 and 0.29447, respectively. It is remarkable that the use of the ADAM training algorithm, which was initially designed for real-valued networks, showed good behavior for training complex-valued networks, making the adaptation described in [33].

Furthermore, the comparison which we propose in this study focuses on demonstrating the ability with simple structures so as not to deal with black box processes [34] that could cloud the objective comparison of these two classes of algorithms. However, we consider it important to assess the use of the transfer learning technique [35]. This would involve building complex-valued structures, which are deeper, and probably, will perform better than the results presented in this research work. In contrast, this would require higher computational costs and longer training times.

Lastly, we believe that the exponential growth of real-valued convolution networks was mainly a result of having access to big enough datasets to train the millions of parameters that form them. This can be verified with the Imagenet challenge [36]. Notwithstanding, the datasets that appeared were all defined in the real number set. Our opinion is that this could stop the growth and development of complex-valued networks, as there were no data that would allow experimentation with the algorithms in this numerical domain. We, therefore, decided to assess the potentiality of the complex-valued convolution networks with datasets in the real number domain, seeking to observe their capacity for this type of task [10].

## 5. Conclusions

Complex-valued networks show better performance for the F1 Score, Precision, Recall, and Specificity metrics in comparison to real-valued networks. This suggests higher potentiality for the classification of melanoma using dermatoscopy images. However, it should be noted that a change of numerical domain must be performed before the real-valued inputs are processed. We made use of the Fourier transform, although it is not the only option available for this task.

In terms of trainable parameters, fair comparison opens the door to building deeper structures that may perform better than those that are presented in this study. It should be noted that the complex-valued networks are defined as even real-valued tensors, but the learning process is performed jointly and not independently.

Complex-valued convolution networks show a limitation from the point of view of theoretical contribution, which is in the scope of the operations that they can carry out in the complex numbers domain. It should be noted that not all the layers that are defined in a real-valued structure can be reproduced for the complex numbers domain. This causes those structures to be not completely comparable. This limitation arose due to the theoretical base that exists for real-valued networks. Further study of these novel algorithms is, however, required. The aim is to find layers that use the information the same way in real-valued structures as in complex-valued structures.

Similarly, complex-valued activation functions are limited in the Hilbert space as the Cauchy–Riemann conditions must be met for the entire space defined. This is a very relevant consideration if we intend to build a complex-valued gradient-based training algorithm. We, therefore, believe that deeper mathematical study is needed so that we can find holomorphic activation functions. This initially limits the possibilities, as only the constant function satisfies this property. The above limitation gives rise to a future alternative of building a non-gradient-based training algorithm [37] or the adaptation (as we have done in this research work) of a real-valued algorithm [33,38] to train complex-valued structures.

For future lines of research, real-valued and complex-valued networks need to be tested for data of a complex nature, i.e., a fair comparison to complement the approach presented herein. This may lead to the generalization of the ability of complex-valued convolution neural networks, transforming them into a universal algorithm for classification problems, regardless of the numerical nature of the input data.

The transfer learning technique must be addressed to work with deeper networks that show higher performance than the state of the art for the classification of the datasets used in this study. Nevertheless, hybrid approaches should equally be considered, i.e., layers totally in parallel, in the real number and complex number domains, which can perform simultaneous feature extractions that can improve the performance of the system studied with lower computational costs.

Once the desired model is obtained, it should be tested in different hospital scenarios. The validated model can be connected to a web interface through a cloud application. This approach will allow the healthcare staff to access easily, and thus they will have artificial intelligence support for the detection of melanoma and heart murmurs. This will be very useful in places with limited access to health care services or where the probability of the disease is high, and a quick and early diagnosis is required to prevent the evolution of the anomalies presented by patients of all ages.

In order to go deeper into the explanation of the high capacity of complex-valued deep learning, it is necessary to carry out a comprehensive study of the learned characteristics, using saliency maps, activation maps, gradient maps, and similar tools to understand in an intuitive way how this novel algorithm performs better than the real-valued deep learning. We consider it very important to highlight that the result of this process will be extensively different compared to the analysis of the real-valued deep learning due to the abstract nature of the complex numbers.

Although complex-valued algorithms are evolving, their use has not spread, as in the case of real-valued algorithms. We can categorically generalize that real-valued algorithms are a particular case of complex-valued algorithms when their imaginary part is zero. However, we consider it very important to know the performance of both approaches (algorithms) under similar operating conditions, i.e., with an equivalent amount of training parameters. Our purpose is to conclude on the higher ability of complex-valued algorithms to extract features, compared with the real-valued algorithms, increasing the discriminative ability in the classification task of the real-valued input data. In this way, we have eliminated the possible bias caused by the difference in the number of trainable parameters. Moreover, we have added a numerical domain conversion step (from real to complex) based on the Fourier transform, although, in the paper, we did not directly conclude the specific reason for its higher capacity for the task of melanoma and heart sound detection. However, our contribution focuses on the fact that we statistically evidenced the superiority of complex-valued convolution networks for the same classification task under equivalent conditions. Our work establishes a comparative precedent of the studied algorithms for disease detection applications on real-valued signals.

## Figures and Tables

**Figure 2:**
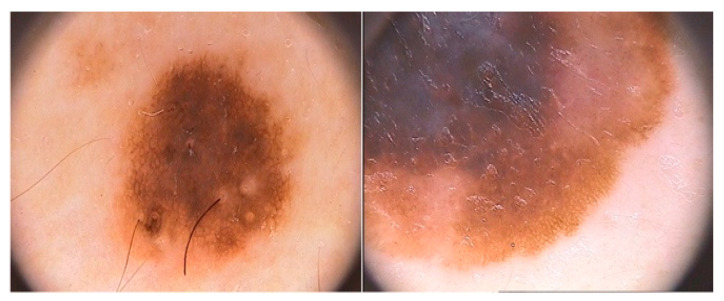
Examples of normal and abnormal images from the PH2 dataset.

**Table 1:** Review of the state of the art in the most representative works related to the use of complex-valued neural networks for classification tasks.

Authors	Dataset Used	Task	Methods	Results
Yue Qi, Qiu Hua Lin, Li Dan Kuang, Wen Da Zhao, Xiao Feng Gong, Fengyu Cong, Vince D. Calhoun [8]	Used 82 resting-state complex-valued fMRI datasets, including 42 SZs and 40 HCs	Classifying schizophrenia patients (SZs) and healthy controls (HCs)	This study proposes a novel framework combining independent component analysis (ICA) and complex-valued convolutional neural networks (CVDL). ICA is first used to obtain components of interest that have been previously implicated in schizophrenia.	- The proposed method shows an average accuracy of 72.65% in the default mode network and 78.34% in the auditory cortex for slice-level classification. - When performing subject-level classification based on majority voting, the result shows 91.32% and 98.75% average accuracy.
Shizhen Hu, Seko Nagae, Akira Hirose [13]	They prepared 7 different concentration samples and measured 30 times for each sample	Glucose concentration estimation	In this paper, an adaptive glucose concentration estimation system is proposed. The system estimates glucose concentration values non-invasively by making full use of transmission magnitude and phase data. The 60–80 GHz frequency band millimeter wave is chosen, and a single output neuron complex-valued neural network (CVNN) is built for adaptive concentration estimation.	- The system shows a good generalization ability to estimate the concentration for unknown samples. It is effective in the estimation of the glucose concentration in the clinically practical range. - The mean squared error (MSE) for the CVNN is 0.011, while the MSE for the RVNN is 0.099.
Joshua Bassey, Xiangfang Li, Lijun Qian [3]	Used 167 publications	Discuss the recent development of CVNNs	A detailed review of various CVNNs in terms of activation function, learning and optimization, input and output representations, and their applications in tasks such as signal processing and computer vision are provided, followed by a discussion on some pertinent challenges and future research directions.	Complex-valued neural networks, compared to their real-valued counterparts, are still considered an emerging field and require more attention and action from the deep learning and signal processing research community.
Yang Ximei [6]	A total of 5 radar data pre-processing approaches were implemented to generate dataset samples, including FFT and STFT	Human-motion classification based on monostatic radar	This thesis proposes three complex-valued convolutional neural networks (CNNs) for human-motion classification based on monostatic radar. The range-time, range-Doppler, range-spectrum-time, and time-frequency spectrograms of micro-Doppler signatures are adopted as the input to CVNNs with different plural-handled approaches. A series of experiments determine the optimal approach and data format that achieves the highest classification accuracy.	- As for 5 radar data formats, range-time and pseudo-Doppler-time have the highest accuracy (92.6% and 87.5%, respectively), followed by range-spectrum-time and range-Doppler (81.3% and 72.3%, respectively). Doppler-time has the worst performance with only 62% accuracy. - Deep neural networks achieve the best classification accuracy on CVNNs, while shallow neural networks do not.
Shubhankar Rawat, K.P.S. Rana, Vineet Kumar [7]	A total of 5232 CXR images from 5856 patients aged 1 to 5 years from Guangzhou Women and Children’s Medical Center, Guangzhou, Guangdong province (China). For this work, out of the 5232 images, only 500 images were considered for MID experimentations, which were randomly selected	Investigate a novel complex-valued convolutional neural network-based model, termed CVMIDNet, for medical image denoising	The model uses residual learning, which learns noise from noisy images and then subtracts it from noisy images so as to obtain clean images. To assess the denoising performance of CVMIDNet, standard image quality metrics, namely, peak signal to noise ratio and the structural similarity index, have been used for 5 different additive white Gaussian noise levels in chest X-ray images. Chest X-ray denoising performance of CVMIDNet was compared with 4 recent state-of-the-art models, namely, BlockMatching and 3D (BM3D) filtering, DnCNN, and Feature-guided Denoising Convolutional Neural Network. (FDCNN), and deep CNN with residual learning.	CVMIDNet was found to be superior. For instance, for a Gaussian noise level of σ = 15, the peak signal-to-noise ratio and structural similarity index values achieved by the CVMIDNet are 37.2010 and 0.9227, respectively, against the 36.2292 and 0.9086, 36.3203 and 0.9139, 35.0995 and 0.9005, 36.1830 and 0.8968, 34.2436 and 0.8874 achieved by BM3D filtering, DnCNN, RVMIDNet, FDCNN, and deep CNN with residual learning, respectively.
Theresa Scarnati, Benjamin Lewis [4]	SAMPLE dataset includes 10 classes with equal numbers of measured and synthetic SARimages: 1366 measured and 1366 synthetic. Total: 2732	They present a survey of several complex neural network techniques as applied to a SAR dataset consisting of military targets	Specifically, they evaluate a multi-channel approach with Deep Complex Networks and SurReal against (i) limited training data and (ii) when the training and testing data exhibit a domain mismatch.	- The SurReal network performs best when trained with measured data, and the multi-channel approach with real and imaginary channels performs best when trained with synthetic data.
Bungo Konishi, Akira Hirose, Ryo Natsuaki [14]	An interferogram around Mt. Fuji observed on 25 November 2010 and 12 April 2011. An interferogram around Shinmoe-dake observed on 14 April 2009 and 30 May 2009	In this paper, they propose complex-valued reservoir computing (CVRC) to deal with complex-valued images in interferometric synthetic aperture radar (InSAR)	They classify InSAR image data by using CVRC successfully with a higher resolution and a lower computational cost, i.e., one hundredth learning time and one-fifth classification time than convolutional neural networks.	CVRC is found applicable to quantitative tasks dealing with continuous values as well as discrete classification tasks with higher accuracy.
Linfang Xiao, Yilong Liu, Zheyuan Yi, Yujiao Zhao, Linshan Xie, Peibei Cao, Alex T L Leong, Ed X Wu [15]	T1w GRE axial brain dataset: 57 and 10 subjects with 200 axial slices extracted from each subject were used for training and testing, respectively	To provide a complex-valued deep learning approach for partial Fourier (PF) reconstruction of complex MR images	They propose a complex-valued deep learning approach with an unrolled network architecture for PF reconstruction that iteratively reconstructs OF sampled data and enforces data consistency. They evaluate their approach for reconstructing both spin-echo and gradient-echo data. They compared the proposed deep learning PF (DL-PF) method to the conventional POCS-PF method.	The proposed method outperformed the iterative POCS PF reconstruction method. It produced better artifact suppression and recovery of both image magnitude and phase details in the presence of local phase changes. Moreover, the network trained on axial brain data could reconstruct sagittal and coronal brain and knee data.
Duan C, Xiong Y, Cheng K, Xiao S, Lyu J, Wang C, Bian X, Zhang J, Zhang D, Chen L, Zhou X, Lou X [16]	SWI data were acquired from 117 participants who underwent clinical brain MRI examinations between 2019 and 2021, including patients with tumor, stroke, hemorrhage, traumatic brain injury, etc.	Propose a deep learning model to accelerate susceptibility-weighted imaging (SWI) acquisition times and evaluate the clinical feasibility of this approach	A complex-valued convolutional neural network (ComplexNet) was developed to reconstruct high-quality SWI from highly accelerated k-space data. ComplexNet can leverage the inherently complex-valued nature of SWI data and learn richer representations by using complex-valued networks.	The average reconstruction time of ComplexNet was 19 ms per section (1.33 s per participant). ComplexNet achieved significantly improved quantitative image metrics compared to a conventional compressed sensing method and a real-valued network with acceleration rates of 5 and 8 (*p* < 0.001). ComplexNet showed comparable diagnostic performance to the fully sampled SWI for visualizing a wide range of pathology, including hemorrhage, cerebral microbleeds, and brain tumors.
Haozhen Li, Boyuan Zhang, Haoran Chang, Xin Liang, Xinyu Gu [5]	CSI dataset generated by COST2100 channel model is used. The training, validation, and testing sets contain 100,000, 30,000, and 20,000 samples, respectively	They present a complex-valued lightweight neural network for channel state information (CSI) feedback named CVLNet	The CVLNet adopts the complex-valued neural network components in a multi-scale feature augmentation encoder and a multi-resolution X-shaped reconstruction decoder with a series of lightweight details.	The experiment results show that the proposed CVLNet maintains the same-level parameters of the encoder with state-of-the-art (SOTA) lightweight networks while outperforming them with at most a 33.4% improvement in accuracy under severe compression rates.

**Table 2:** Summary table of the most relevant characteristics of the ISI2017, PH2, and Pascal datasets.

Name of the Database	Normal Data	Abnormal Data	Data Type	Associated Illness
ISIC2017	1621	374	Dermatoscopy image	Melanoma
PH2	160	40	Dermatoscopy image	Melanoma
PASCAL	320	141	Sounds/Scalogram	Heart murmurs

**Table 3:** Experiment design for this research work.

Structure/Database	ISIC2017	PH2	PASCAL
Complex-valued structure	Accuracy, F1 Score, Precision, Recall, Sensitivity, Specificity	ibidem	Ibidem
Real-valued structure	ibidem	ibidem	Ibidem

**Table 4:** Table of hyperparameters used for the complex/real-valued convolution networks.

Hyperparameter	Complex-Valued	Real-Valued
Activation function	Complex Relu	Relu
Learning Rate	0.001	0.001
Optimizer	ADAM with Complex Correction	ADAM

**Table 5:** Number of parameters in the networks studied.

Layer	Amount of Parameters Complex-Valued	Amount of Parameters Real-Valued
Conv1	71,940	290,400
Conv2	186,608	285,144
Conv3	725,760	1,492,992
Fully Connected 1	169,600	359,552
Fully Connected 2	1200	1200
Fully Connected 3	500	500
Output	2	2

**Table 6:** Results obtained with the complex-valued convolution network studied for the ISIC2017 dataset.

Structure/Metric	Fold	F1 Score	Precision	Recall/Sensitivity	Accuracy	Specificity
Complex-Valued Convolution Neural Networks	1	0.90410	0.89411	0.914328	0.77889	0.73888
	2	0.91490	0.90734	0.922586	0.76382	0.74650
	3	0.93140	0.93938	0.923584	0.79899	0.75860
	4	0.91270	0.91895	0.906528	0.80402	0.73957
	5	0.92270	0.94316	0.903088	0.79397	0.78863
	6	0.93140	0.93279	0.930083	0.79397	0.74694
	7	0.89580	0.89813	0.893478	0.77387	0.75531
	8	0.90550	0.92049	0.890943	0.76884	0.75336
	9	0.91700	0.90336	0.931074	0.80402	0.74156
	10	0.93320	0.94119	0.925291	0.82412	0.76313
Max Complex		0.93320	0.94316	0.931074	0.93107	0.78863
Min Complex		0.89580	0.89411	0.890943	0.89094	0.73888
Mean Complex		0.91690	0.91989	0.914098	0.91409	0.75325
Normality Test/*p*-value		0.55702	0.28137	0.09199	0.70872	0.21898

**Table 7:** Results obtained with the real-valued convolution network studied for the ISIC2017 dataset.

Structure/Metric	Fold	F1 Score	Precision	Recall/Sensitivity	Accuracy	Specificity
Real-Valued Convolution Neural Networks	1	0.86960	0.86078	0.87854	0.66834	0.66298
	2	0.88730	0.90188	0.87316	0.69347	0.66127
	3	0.87180	0.86158	0.88229	0.67337	0.64810
	4	0.86750	0.86807	0.86694	0.73869	0.67762
	5	0.88280	0.90251	0.86399	0.68342	0.63900
	6	0.87890	0.87545	0.88247	0.68342	0.63624
	7	0.87910	0.87691	0.88128	0.66332	0.67711
	8	0.86120	0.87116	0.85153	0.69849	0.67995
	9	0.87420	0.88535	0.86323	0.66834	0.67312
	10	0.88780	0.88409	0.89156	0.65829	0.67185
Max		0.88780	0.90251	0.89156	0.73869	0.67995
Min		0.86120	0.86078	0.85153	0.65829	0.63624
Mean		0.87600	0.87878	0.87350	0.68291	0.66272
Normality Test/*p*-value		0.10060	0.32868	0.77467	0.07353	0.11563

**Table 8:** Means comparison hypothesis test for dataset ISIC 2017.

Metric	Student’s *t*-Test Comparison of Means—*p*-Value
F1 Score	0.00001
Precision	0.00004
Recall	0.00002
Accuracy	0.00001
Specificity	0.00001

**Table 9:** Results obtained with the complex-valued convolution network studied for the PH2 dataset.

Structure/Metric	Fold	F1 Score	Precision	Recall/Sensitivity	Accuracy	Specificity
Complex-Valued Convolution Neural Networks	1	0.90909	0.93750	0.88235	0.88235	0.66667
	2	0.90909	0.93750	0.88235	0.88235	0.66667
	3	0.86667	0.92857	0.81250	0.81250	0.75000
	4	0.87500	0.93333	0.82353	0.82353	0.66667
	5	0.84615	0.91667	0.78571	0.78571	0.83333
	6	0.89655	0.92857	0.86667	0.86667	0.80000
	7	0.90323	0.93333	0.87500	0.87500	0.75000
	8	0.91429	0.94118	0.88889	0.88889	0.50000
	9	0.86667	0.92857	0.81250	0.81250	0.75000
	10	0.88889	0.92308	0.85714	0.85714	0.83333
Max Complex		0.91429	0.94118	0.88889	0.85000	0.83333
Min Complex		0.84615	0.91667	0.78571	0.80000	0.50000
Mean Complex		0.88756	0.93083	0.84866	0.83000	0.72167
Normality Test/*p*-value		0.71070	0.14828	0.36900	0.00017	0.14913

**Table 10:** Results obtained with the real-valued convolution network studied for the PH2 dataset.

Structure/Metric	Fold	F1 Score	Precision	Recall/Sensitivity	Accuracy	Specificity
Real-Valued Convolution Neural Networks	1	0.81818	0.90000	0.75000	0.80000	0.87500
	2	0.82353	0.87500	0.77778	0.70000	0.00000
	3	0.81250	0.86667	0.76471	0.70000	0.33333
	4	0.81250	0.86667	0.76471	0.70000	0.33333
	5	0.76923	0.83333	0.71429	0.70000	0.66667
	6	0.81250	0.86667	0.76471	0.70000	0.33333
	7	0.81250	0.86667	0.76471	0.70000	0.33333
	8	0.80000	0.85714	0.75000	0.70000	0.50000
	9	0.78571	0.84615	0.73333	0.70000	0.60000
	10	0.81250	0.86667	0.76471	0.70000	0.33333
Max		0.82353	0.90000	0.77778	0.80000	0.87500
Min		0.76923	0.83333	0.71429	0.70000	0.00000
Mean		0.80592	0.86450	0.75489	0.71000	0.43083
Normality Test/*p*-value		0.21406	0.05052	0.15922	0.00001	0.31439

**Table 11:** Mean comparison hypothesis test for dataset PH2.

Metric	Student’s *t*-Test Comparison of Means—*p*-Value
F1 Score	7.71763 × 10^8^
Precision	1.13570 × 10^7^
Recall	6.08852 × 10^6^
Specificity	4.11085 × 10^3^

**Table 12:** Results obtained with the complex-valued convolution network studied for the Pascal dataset.

Structure/Metric	Fold	F1 Score	Precision	Recall/Sensitivity	Accuracy	Specificity
Complex-Valued Convolution Neural Networks	1	0.82540	0.89655	0.76471	0.76596	0.76923
	2	0.87273	0.92308	0.82759	0.84783	0.88235
	3	0.80702	0.88462	0.74194	0.76087	0.80000
	4	0.88889	0.92308	0.85714	0.86957	0.88889
	5	0.81967	0.89286	0.75758	0.76087	0.76923
	6	0.84211	0.88889	0.80000	0.80435	0.81250
	7	0.83582	0.90323	0.77778	0.76087	0.70000
	8	0.86792	0.92000	0.82143	0.84783	0.88889
	9	0.83077	0.90000	0.77143	0.76087	0.72727
	10	0.83582	0.90323	0.77778	0.76087	0.70000
Max Complex		0.88889	0.92308	0.85714	0.86957	0.88889
Min Complex		0.80702	0.88462	0.74194	0.76087	0.70000
Mean Complex		0.84261	0.90355	0.78974	0.79399	0.79384
Normality Test/*p*-value		0.22495	0.58013	0.48847	0.00280	0.18135

**Table 13:** Results obtained with the real-valued convolution network studied for the Pascal dataset.

Structure/Metric	Fold	F1 Score	Precision	Recall/Sensitivity	Accuracy	Specificity
Real-Valued Convolution Neural Networks	1	0.74510	0.82609	0.67857	0.72340	0.78947
	2	0.76923	0.83333	0.71429	0.73913	0.77778
	3	0.76667	0.85185	0.69697	0.69565	0.69231
	4	0.78788	0.83871	0.74286	0.69565	0.54545
	5	0.75862	0.84615	0.68750	0.69565	0.71429
	6	0.75000	0.82759	0.68571	0.65217	0.54545
	7	0.80702	0.85185	0.76667	0.76087	0.75000
	8	0.76471	0.83871	0.70270	0.65217	0.44444
	9	0.77966	0.85185	0.71875	0.71739	0.71429
	10	0.80702	0.85185	0.76667	0.76087	0.75000
Max		0.80702	0.85185	0.76667	0.76087	0.78947
Min		0.74510	0.82609	0.67857	0.65217	0.44444
Mean		0.77359	0.84180	0.71607	0.70930	0.67235
Normality Test/*p*-value		0.06181	0.17432	0.41637	0.36105	0.05332

**Table 14:** Mean comparison hypothesis test for Pascal Dataset dataset.

Metric	Student’s *t*-Test Mean Comparative
F1 Score	4.0131 × 10^9^
Precision	1.4683 × 10^4^
Recall	5.0077 × 10^6^
Specificity	0.01450

**Table 15:** Results of the Mann–Whitney U hypothesis test.

Metric	Dataset	Test Executed	*p*-Value
Accuracy	PH2	U	0.00134
Accuracy	Pascal	U	0.02377

## Data Availability

Not applicable.
